# COPD burden on sexual well-being

**DOI:** 10.1186/s12931-020-01572-0

**Published:** 2020-11-25

**Authors:** M. Zysman, J. Rubenstein, F. Le Guillou, R. M. H. Colson, C. Pochulu, L. Grassion, R. Escamilla, D. Piperno, J. Pon, S. Khan, C. Raherison-Semjen

**Affiliations:** 1 Univ-Bordeaux, Centre de Recherche Cardio-Thoracique de Bordeaux, U1045, CIC 1401, 33604 Pessac, France; 2 Service Des Maladies Respiratoires, Hôpital Haut-Lévèque CHU Bordeaux, 33604 Pessac, France; 3 Santé Respiratoire France, 115 rue Saint Dominique, 75007 Paris, France; 4 L’Association Interdisciplinaire Post Universitaire de Sexologie, Toulouse, France; 5 Service de Pneumologie CHU Toulouse, Toulouse, France; 6 Service de Psychiatrie CHU Toulouse, Toulouse, France; 7 U1219 Inserm, ISPED, University of Bordeaux, Bordeaux, France; 8 Service Des Maladies Respiratoires, CHU Bordeaux, Université de Bordeaux, U1219 EpiceneBordeaux, France

**Keywords:** COPD, Sexual health, Well-being, Quality of life

## Abstract

**Background:**

Sexual function is often affected in patients suffering from chronic diseases especially chronic obstructive pulmonary disease (COPD). However, the effect of COPD on sexual satisfaction is underappreciated in clinical practice. The aim of this study is to evaluate the impact of COPD on patient’s sexuality and the explanatory variables of sexual dissatisfaction.

**Methods:**

Questionnaires were emailed to participants and they submitted their responses on the Santé Respiratoire France website. Data about sexual well-being (Arizona Sexual Experience Scale, ASEX), Quality of life (VQ11), anxiety, depression (Hospitalized anxiety and depression, HAD) and self-declared COPD grade were collected.

**Results:**

Seven hundred and fifty one subjects were included and were characterized as follows: women—51%, mean age—61 years, in a couple—62% and 70%—retired. Every grade of COPD was represented. Out of 751 participants, 301 participants (40%) had no sexual activity and 450 (60%) had sexual activity. From the 450 participants, 60% needed to change their sexual life because of their disease (rhythm, frequency and position). Subjects often used medications to improve sexual performance (43% used short-acting bronchodilator and 13% -specific erectile dysfunction drugs). ASEX questionnaire confirmed patients’ dissatisfaction (diminution of sexual appetite for 68% and sexual desire for 60%) because of breathlessness and fatigue. Eighty one percent of the responders had an altered quality of life (VQ11 mean score 35) and frequent suspected anxiety or depression (HAD mean score 10.8). Ninety percent declared that sexual dysfunction had never been discussed by their doctors, while 36% of patients would have preferred to undergo a specialized consultation.

**Conclusion:**

Sexual dysfunction is frequent among COPD patients and leads to an altered well-being, however being a cultural taboo, it remains frequently neglected. Sexual guidance should be a part of patient’s consultations improve quality of sexual life.

## Background

Sexual function is often affected in individuals living with chronic illness, and multiple comorbidities increase the likelihood of sexual dysfunction. The World Health Organization (WHO) has defined sexual health as a state of physical, mental and social well-being in relation to sexuality [1]. Good sexual health requires a positive and respectful approach to sexuality and sexual relationships, as well as the possibility of having pleasurable and safe sexual experiences, free of coercion, discrimination and violence. It also requires freedom from organic disorders and diseases that interfere with sexual and reproductive functions. However, chronic obstructive pulmonary disease (COPD) is progressive disorder characterized by airflow limitation that is not fully reversible, and is often accompanied by decreased quality of life (QoL) [2]. Sexual function is a QoL aspect that is markedly affected in subjects with COPD [3–7] as the accompanying respiratory or physical symptoms, such as dyspnea, weakness, fatigue and reduced physical activity have an adverse effect on sexual activity [8–10].

Despite these detrimental consequences, the effect of COPD on sexual satisfaction is underappreciated in clinical practice, partly because patients often feel uncomfortable talking about their sexuality with healthcare providers [3, 4, 7, 11] but also because healthcare providers are reluctant to address these issues.

The primary aim of this study was to evaluate the disease’s impact on patients’ sexuality among subjects living with COPD and the explanatory variables of sexual dissatisfaction.

## Methods

### Questionnaire

Online survey was assessed from March to May 2019 through the association Santé Respiratoire France (https://sante-respiratoire.com). Four pulmonologists, one sexologist, one expert patient and one psychiatrist developed the questionnaire. The following data were collected at inclusion: demographic characteristics (age, gender, marital or relationship status, occupation, living area: urban, suburban, rural), smoking history, breathlessness and exacerbations etc.).

A detailed description of the questionnaires is available in Additional file 1.

#### Arizona sexual experience scale (ASEX)

Sexual experience was assessed using the 4-item Arizona Sexual Experience Scale (ASEX-French version), owing to its reliability, validity and easy administration [12]. It allows for the assessment of four major global aspects of sexual dysfunction: sexual appetite/ drive, sexual desire/ arousal, penile erection/vaginal lubrication and satisfaction from orgasm. Higher scores indicate poor sexual function [12].

#### VQ11

Quality of life was evaluated by VQ11 which is intended to assess diverse components of quality of life (functional: 3 items, psychological: 4 items, social: 4 items), through 11 questions, with a rating from 1 to 5, with higher scores indicating decreased quality of life [13, 14].

#### Hospitalized anxiety and depression (HAD)

HAD scale allows for the detection of depression and/or anxiety through 14 items rated from 0 to 3, based on symptom frequency (higher frequency equating to higher scores). A score of 11 or more indicates probable presence of an altered mood [15].

#### Fatigue severity scale (FSS)

The FSS is a questionnaire with nine questions estimating the fatigue severity in different situations during the past week. Grading ranges from 1 (strong disagreement) to 7 (strong agreement) where the final score is the mean value of the nine items, and a score ≥ 4 is interpreted as fatigue [16]. FSS scale has been validated in COPD [17, 18].

Once the questionnaire was completed and tested, it was then emailed to every member of BPCO association. Followed by completion of the survey data by anonymous volunteers, to respect the clause: “strengthening the reporting of observational studies in epidemiology” (STROBE) statement [19]. COPD was validated by a spirometry and by a physician-diagnosis.

### Mapping psychological profiles

We carried out a typological analysis to split the studied population into distinct homogeneous groups depending on their responses to the various questionnaires. We conducted latent class and profile analyses of CODP burden and respiratory assistance (dyspnea, fatigue) and psychological symptoms (depression and anxiety) to identify distinct classes (subgroups) of symptom profiles [20]. Patients were assigned a probability of being in each of the identified classes with the goal of creating a model that uniquely assigned a subject to a given class (e.g. Pr(ClassA) = 1.0; Pr(ClassB) = 0.0), or at minimum, provided a distinctively high probability to a given class versus all others (e.g. 0.95 versus 0.05). Model fit was evaluated using information criteria fit indices (Bayesian Information Criterion, BIC and Akaike’s Information Criterion, AIC); and low values indicate model parsimony [21]. We also used other criteria to identify a meaningful fit of model and data, and these included class interpretability (the extent to which additional classes provided unique information), class prevalence (preferring classes with at least 2% of the sample for improved replicability), and entropy (a measure of classification based on posterior probability values, with higher values representing better classification). We used analysis of variance and Chi-Square statistical analysis, which provides analysis of dependencies between data, establishing a hierarchical relationship, and displaying them in a two-dimensional graphical form. The two main factorial axes identified were the psychological state (ranging from good mental health to worsened mental health) and the need for respiratory assistance (starting from no assistance to respiratory assistance such as oxygen or non-invasive ventilation).

### Statistical analysis

The calculation of the sample was based on the sampling strategy without experimental treatment. The sample size was calculated using the following formula: minimal sample size to obtain significant results for an event and a fixed level of risk: n = 100 X t^2^ × p × (1-p) / m^2^n, with t: Confidence level, the standard value for the 95% confidence level will be 1.96, p: estimated proportion of the population with the characteristic of interest, m: Margin of error (usually set at 5%). Previous studies indicate that sexual dissatisfaction affects between 67.7% and 81% of COPD patients. Thus, for an event with a 60% probability of occurrence, and taking a 95% confidence level and a 5% margin of error, the sample size should be: n = 100 × 1.96^2^ × 0.4 × 0.6 / 0.05^2^ = 368.79. We should have at least 369 subjects and we included more than 700. Qualitative variables were described in numbers and percentages. Quantitative variables were described by mean and standard deviation or median and inter-quartile range. Missing data are reported in the tables. Differences in clinical characteristics were assessed using chi-square tests or Fisher’s exact tests, as appropriate, for discrete variables, and Wilcoxon or Kruskal–Wallis tests for quantitative variables. Relationships between different variables were examined with the Pearson correlation test. A value of p ≤ 0.05 was considered statistically significant.

The analysis of the results was carried out with Graph Pad Prism® statistical software.

## Results

### Responders

Seven hundred and fifty one patients responded to the survey. Participants were predominantly women (51% of patients) with a mean age of 61 years old and 62% of them lived as a couple. The majority of the participants were retired (70%). Every COPD grade was represented and one third of responders received oxygen. Only a quarter of the responders lived in an urban setting. Clinical characteristics are reported in Table 1.

**Table 1:** Characteristics of the studied population

Variables	Subjects
N	751
Age (years)	61 [24–91]
Gender (male)	368 (49%)
Living as a couple	466 (62%)
Living area	
Urban	197 (26%)
Suburban	233 (31%)
Rural	317 (42%)a
Socioprofessional categories	
Farmer	4 (0.5%)
Craftsperson, storekeeper, company head	66 (8.8%)
Executive	123 (16.4%)
Employee	267 (35.6%)
Inactive/non-working	70 (9.3%)
Worker	72 (9.6%)
Technician	149 (19.8%)
FEV_1_ (%)	
FEV1 ≥ 80%	52 (7%)
50% < FEV1 ≤ 80%	225 (30%)
30% < FEV1 ≤ 50%	278 (37%)
FEV1 ≤ 30%	173 (23%)
Number of moderate to severe exacerbations in the previous year	1.95
Daily inhaled therapy	661 (88%)
Oxygenotherapy	233 (31%)
Ventilation (NIV or CPAP)	210 (28%)
At least one comorbidity	481 (64%)
ASEX	
Sexual appetite	
Low	368 (49%)
Absent	143 (19%)
Sexual desire	
Low	330 (44%)
Absent	120(16%)b
Erectile dysfunction	451 (60%)
VQ11	34,8 [29.8–37.1]c
HAD	
HAD-A	10 [8, 9]
HAD-D	8 [5, 6]d
Anxiety HAD A ≥ 10	340 (45%)
Depression HAD D ≥ 10	210 (28%)
FSS	4.67 [3.1–6.24]e

Missing data: a = 4, b = 114, c = 5, c = 103, e = 96

*ASEX* Arizona Sexual Experience Scale, *FSS* Fatigue severity scale *HAD* hospital anxiety and depression scale, VQ11. Data are provided as median [Q1–Q3] or n (%), as appropriate

### Sexual activities

Forty percent had no sexual activity among which, 19% lived as a couple. From the remaining 60%, 74% had to change their rhythm, 64% their frequency and 64% their position. Two thirds of the participants were dissatisfied with their current and expected sexual function and 13% declared that they used specific medications to improve their sexual performance. Forty three percent used routinely (17%) or occasionally (26%) a short-acting bronchodilator before sexual activity. Expressed reasons of sexual dissatisfaction were breathlessness, fatigue and physical problems. Men were more often dissatisfied with their current and expected sexual function (66% of men *vs* 53% of women, p = 0.0002), however it did not translate into more medication, especially bronchodilators (which respectively concerned 28% of men and 22% of women). Besides, the level of sexual dissatisfaction (assessed by the following evaluation: “improved, stable, worsened”) was unrelated to age (p = 0.391) or gender (p = 0.92, Table 2).

**Table 2:** Characteristics of the studied population according to sexual satisfaction

	Improved	Stable	Worsened	P
Gender Male/female	3%/4%	29%/29%	68%/67%	0.92
Age 24–49/50–59/60–69/ > 70 (years)	5%/3%/5%/1%	31%/25%/34%/26%	64%/72%/61%/73%	0.391
Living as a couple (Yes/no)	4%/3%	29%/30%	67%/67%	0.978
Occupation (Yes/no)	2%/5%	32%/28%	66%/67%	0.417

Data are provided as %. Differences in clinical characteristics were assessed using chi-square tests

**Fig. 1:**
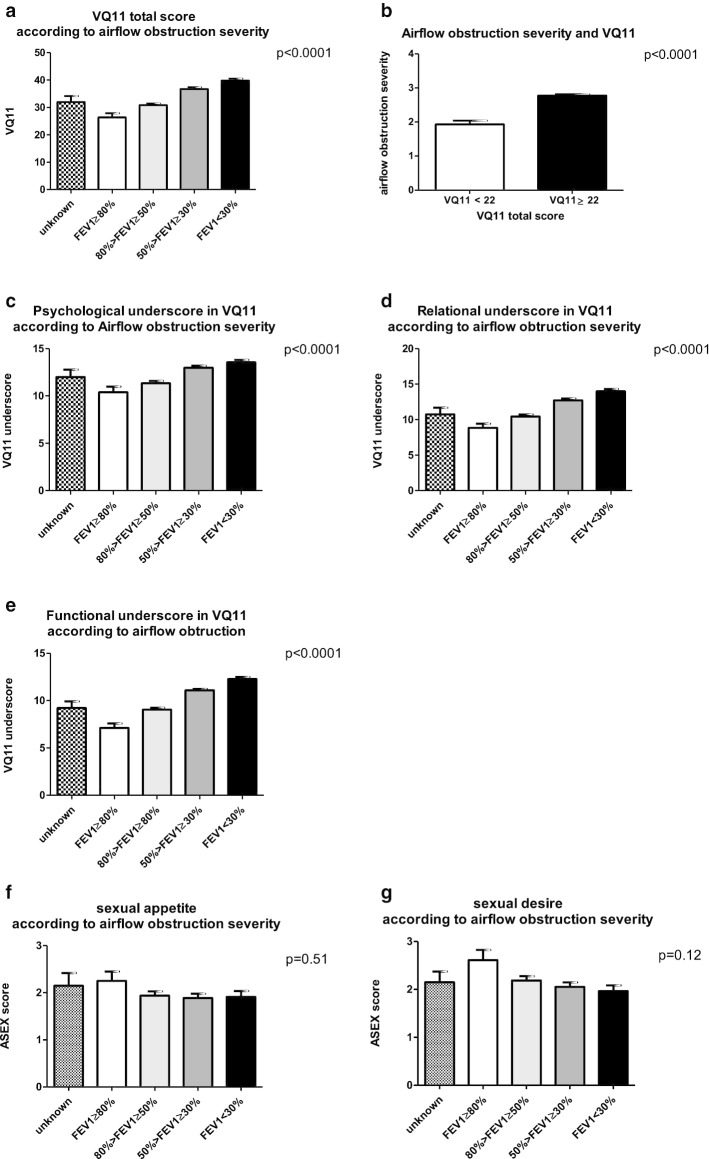
Quality of life, assessed by VQ11 and sexual satisfaction, assessed by ASEX score according to airflow obstruction, assessed by FEV1. **a** VQ11 total score according to airflow obstruction, assessed by FEV1. **b** airflow obstruction according to VQ11 threshold (at 22 points). **c** VQ11 psychological underscore according to airflow obstruction, assessed by FEV1. **d** VQ11 relational underscore according to airflow obstruction, assessed by FEV1. **e** VQ11 functional underscore according to airflow obstruction, assessed by FEV1. **f** Sexual appetite (ASEX) according to airflow obstruction, assessed by FEV1. **g** Sexual desire (ASEX) according to airflow obstruction, assessed by FEV1

ASEX score revealed an altered sexual appetite: low for 49% of the subjects and absent for 19%. Sexual desire was compromised in 44% of the subjects and 16% lost all desire. However, the deterioration of sexual appetite or sexual desire, assessed with ASEX questionnaire, is not linked to airflow obstruction (Fig. 1f, g). Subjects with COPD had to make adaptations to compensate for their diminishing activity tolerance, increasing dyspnea, role and sexual self-image, anxiety, and increased need for communication. Self-reported libido loss and reduced sexual performance worsened over time for 67% of the responders.

### Quality of life and depression and anxiety

Based on the cut-off of 22 for VQ11 score, quality of life was altered among responders with a mean score of 34.8. The increase in airflow obstruction severity was inversely proportional to the quality of life, as assessed with VQ11 augmentation (Fig. 1a). Impaired quality of life (defined as VQ11 ≥ 22) is significantly more frequent among responders with a higher airflow obstruction (Fig. 1b). Specific underscores, including psychological, functional and relational components of quality of life, indicate that every aspect of life is impaired and an increasing with airflow obstruction further impairs the quality of life (Fig. 1c-e).

**Fig. 2:**
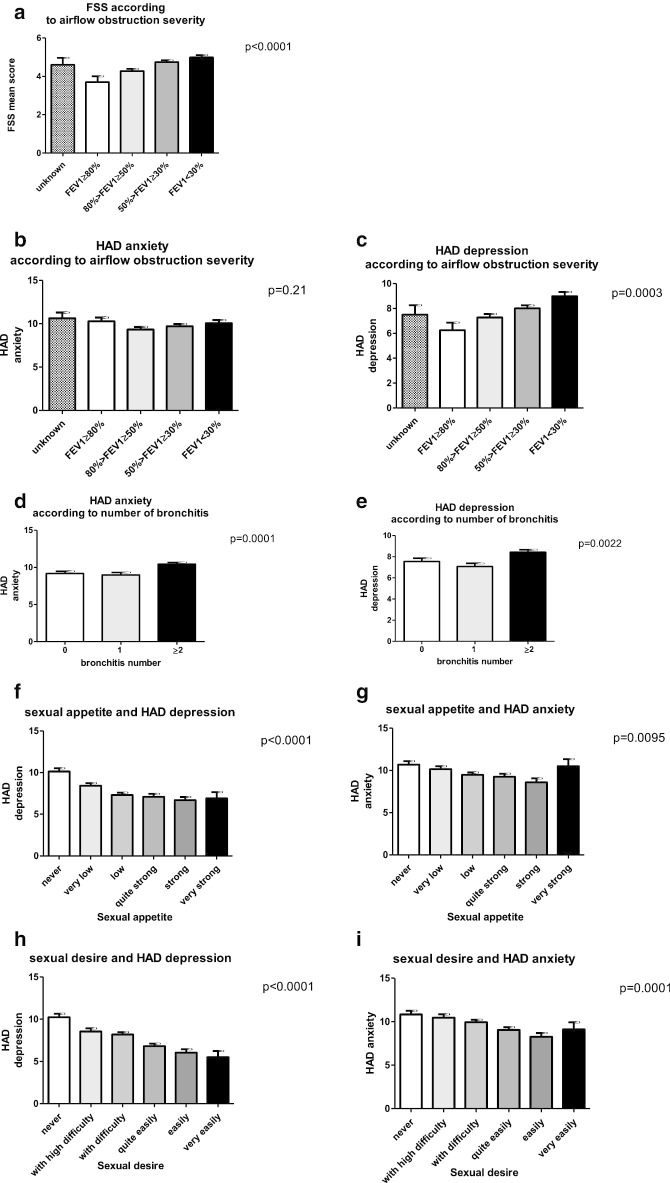
Mental disorder such as anxiety and depression are associated with COPD severity in the responders. **a** FSS total score according to airflow obstruction, assessed by FEV1. **b** Anxiety, assessed with HAD-anxiety subscore according to airflow obstruction, assessed by FEV1. **c** Depression, assessed with HAD-depression subscore according to airflow obstruction, assessed by FEV1. **d** Anxiety, assessed with HAD-anxiety subscore according to the number of bronchitis. **e** Depression, assessed with HAD-depression subscore according to the number of bronchitis. **f** Sexual appetite (ASEX) according to depression, assessed by HAD-depression. **g** Sexual appetite (ASEX) according to anxiety, assessed by HAD-anxiety. **h** Sexual desire (ASEX) according to depression, assessed by HAD-depression. **i** Sexual desire (ASEX) according to anxiety, assessed by HAD-anxiety

Mental disorders such as anxiety and depression were associated with COPD severity in the responders. A mean score of 4.6 on the FSS scale translates into a depressive tendency in patients and FSS mean score increases with airflow obstruction severity, as shown in Fig. 2a. Similarly, HAD scale found high proportions of patients with suspected anxiety or depression with a mean score of 10 and 8 for HAD-A and HAD-D respectively. HAD mean score for depression underscore increased with airflow severity (Fig. 2c) but the same was not reflected in HAD mean score for anxiety (Fig. 2b). However, both HAD scores for anxiety and depression increased when responders had at least two episodes of bronchitis in the previous year (Fig. 2d, e).

Mental disorders are also associated with sexual dissatisfaction. Higher depression as assessed by HAD underscore for depression was strongly indicative of decreased sexual appetite and to a lesser extent anxiety as assessed with HAD underscore for anxiety (Fig. 2f, g). Inversely, a higher impairment of sexual desire caused an increase in depression and anxiety (Fig. 2h, i).

### Sexuality remains taboo

Discussion of sexual issues remains a cultural taboo. Ninety percent of the responders declared that sexual dysfunction had never been discussed by doctors (general practitioners or pulmonologists) and only 6% had a specialized consultation whereas 36% of them declared that they would have liked a specialized consultation with a sexologist. Twenty one percent would be willing for an online consultation.

### Psychological profiles

**Fig. 3:**
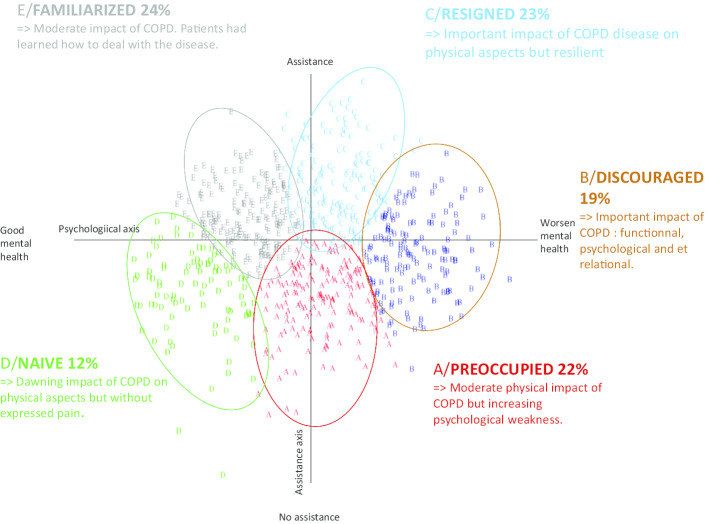
Mapping of individuals gave a representation of their psychological profile

We carried out typological analyses to split the population studied into distinct homogeneous groups depending on their responses to the different questionnaires. This allowed us to combine the individuals into distinct homogeneous groups. We were able to isolate five behavioral profiles: Group A Preoccupied (22%), Group B Discouraged (19%), Group C Resigned (23%), Group D Naive (12%) and Group E Familiarized (24%). The map in Fig. 3 provides a pictorial representation of these 5 profiles.

The patients in group A were predominantly female, active, less than 60 years old, with recently diagnosed and non-severe COPD. These patients had a moderate physical impact of COPD, suffering from symptoms of depression and anxiety.

The patients in group B were also predominantly female, but polymorbid, suffering from severe COPD requiring respiratory assistance. They can no longer, or with difficulty, maintain a sexual life, and have important psychological symptoms leading to social isolation. The physical impact of COPD, but above all psychological and relational impact within this subgroup was clearly evident (extreme right-hand side of the graph).

The patients in group C were older than 70, with a long history of severe COPD, requiring daily ventilation. The degradation of their emotional and sexual life could be attributed to inactivity, breathlessness and substantial fatigue. They suffered mainly from physical symptoms, which explain their position at the top of the graph. However, they displayed only minor psychological symptoms.

The patients in group D were young and active, with recently diagnosed, non-severe COPD not requiring any respiratory assistance. Their sexual life was unaffected and for the disease did not require any life modification. Their social and emotional lives were normal. This group is situated at the lower left quadrant of the graph, displaying low limitations on the two axes.

Finally, the patients in group E were elderly, suffered from a longstanding moderate COPD. They remained active despite breathlessness. They were not worried or anxious, and tried to adapt according to their respiratory capacity. These were patients with physical symptoms largely, albeit of a moderate nature.

## Discussion

Till date, our study is the largest one to focus exclusively on sexual health in COPD patients. As reported previously, more than two thirds of patients with COPD had sexual dissatisfaction [11]. In a Taiwanese cohort study of 57,928 participants, patients with COPD had 1.88-fold more sexual problems than patients without COPD [22]. Most of the previous studies have focused on male impotence, and only two studies included women with COPD [8–10], however our study indicates a dissatisfaction related to sexual activity within both genders. These findings suggest that active care and attention should be directed to the sexual function and sexual satisfaction of COPD female patients as well as male patients. We also found that sexual activity of COPD patients in our cohort was related to the same factors that are of importance in a healthy population, namely: gender, partner status (single or in couple) and age [4, 10, 23, 24].

Sexual dysfunctions are highly prevalent in both sexes, ranging from 10 to 52% of men and 25% to 63% of women [25, 26]. Sexual dysfunction is usually strongly related to age and health status [27]. Several studies have examined the impact of chronic diseases on sexual dysfunction and erectile dysfunction has been described as a “sentinel symptom” of several chronic diseases (hypertension, diabetes, ischemic heart disease) [3]. For example, hypertension significantly increases sexual dysfunction (odds ratio = 2.789, p = 0.002) compared with the normotensive group [28]. However the effect of intensive blood pressure lowering is moderate (p for interaction = 0.0016, [29]). Diabetes is also one of the most frequent organic causes of sexual dysfunction affecting around 20 to 36% of subjects [30]. COPD and cardiovascular or metabolic diseases also seem to affect sexual function. This could be owing to the shared common pathways between the mechanisms underlying sexual dysfunction and the mentioned. Considering biological mechanisms; high levels of inflammatory markers such as TNFα have been described in both COPD and sexual dysfunction [6], and chronic hypoxia, systemic/endothelial inflammation, hormonal imbalances (hypogonadism or lower testosterone levels) have also been considered to be possible contributing factors [31].

Moreover, the link between sexual dysfunction and COPD may be the consequence of a decreased physical activity in patients leading to deconditioning. Experience of sexual dysfunction is more likely among women and men with poor physical health [24]. In agreement with this hypothesis we found that airflow obstruction severity is associated with sexual disability, although we did not perform exercise test among participants to clearly establish a link between physical inactivity and sexual dysfunction.

Nevertheless, respiratory or physical symptoms, such as dyspnea, cough, weakness, and reduced physical activity or fear of breathing difficulty, were reported to affect sexual activity, lowering the QoL of responders and COPD patients in general. For many patients, the libido loss triggers a sense of shame, which can reduce their self-esteem and cause depression. This study also confirms that individuals with COPD are at higher risk for developing depression [32] and subsequent worsening of sexual dysfunction.

Although doctors are reluctant to address the topic of sexuality in practice, our data suggest that patients want their doctors to address these concerns and provide resources to them. Despite the high incidence of sexual dissatisfaction among COPD patients, only 6% had talked about sexuality with a specialist. However, many clinicians may lack some of the basic skills needed to confidently address these concerns [33]. Additionally, doctors should be proactive in initiating conversations on sexual issues to fill this gap. In practice, evaluation of quality of life in patients with COPD is currently being taking into account; however doctors and generally healthcare professionals need to integrate sexual well-being as an important component of quality of life.

This study has some limitations. The questionnaire results are, by definition, declarative, which may be a source of bias. Nevertheless, in order to alleviate this limitation we compared responders’ clinical characteristics to those in large international COPD cohort, such as ECLIPSE. Airflow obstruction assessed by FEV1 is similar in our population as compared to ECLIPSE cohort; GOLD stages 2, 3 and 4 affected 30, 37 and 23% in our population and 44, 42 and 14% in ECLIPSE [34]. Similarly, exacerbation rates in the previous year were also similar with 1.21 in ECLIPSE [35] as compared to 1.95 in our population. The same repartition of airflow obstruction stages was observed with French COPD cohort initiative BPCO or PALOMB [36, 37] assuring a better reproducibility of our data. Another limitation is the lack of objective parameters to measure COPD severity such as centralized spirometry. Responders include patients coming from both urban and rural and a wide range of—occupational categories but unfortunately we did not collect data about their level of education. We also have no data about history of sexual dysfunction, hence unable to ascertain whether sexuality difficulties occurred prior to or after COPD diagnosis. Co-morbidities frequently associated with COPD [32], can also be involved in sexual dysfunction such as hypertension, chronic cardiovascular disease diabetes and obesity or sedentary lifestyle [38] were also not recorded. It is therefore difficult to determine the attributable share of COPD in sexual dysfunction. Future studies should examine changes in patient’s sexual disability over time according to COPD progression.

## Conclusions

Sexual dysfunction is frequent among COPD patients and often neglected. Healthcare workers should strive to discuss sexual problems with patients suffering from COPD and offer sexual guidance as a means to improve quality of sexual life. Particular attention should be paid to women sexual dissatisfaction which is often under-estimated.

## Supplementary information

**Additional file 1:** COPD burden on sexual well-being, RERE-D-20–00192, R2.

## Data Availability

The datasets used and analyzed during the current study are available from the corresponding author on reasonable request.

## Abbreviations

ASEX: Arizona sexual experience scale
BMI: Body mass index
COPD: Chronic obstructive pulmonary disease
FEV1: Forced expiratory volume in one second
GOLD: Global initiative for obstructive lung disease
HAD: Hospital anxiety and depression scale
mMRC: Modified Medical Research Council
OSAS: Obstructive sleep apnea syndrome
