# Supplementary material for: COPD burden on sexual well-being
Source: Respir Res. 2020 Nov 25;21:311. doi: 10.1186/s12931-020-01572-0 (PMC7687801; doi:10.1186/s12931-020-01572-0)
Supplement: Supplementary file 1 — Additional file 1: COPD burden on sexual well-being, RERE-D-20–00192, R2. [file 12931_2020_1572_MOESM1_ESM.doc]

**Supplement data: COPD burden on sexual well-being, RERE-D-20-00192, R2**

A detailed description of the questionnaires

Arizona Sexual Experience Scale (ASEX )

Sexual experience was assessed using the 4-item French version of Arizona Sexual Experience Scale (ASEX), which is reliable, valid and is easy to administer [1]. The ASEX is designed to assess four major global aspects of sexual dysfunction: sexual appetite/ drive, sexual desire/ arousal, penile erection/vaginal lubrication and satisfaction from orgasm. It is appropriate for patients of any sex and sexual orientation, with or without partners. Responses are scored on a 1-6 Likert scale with a potential range of 5-30. Higher scores indicate worsening of sexual function and it has been demonstrated to have excellent reproducibility [1]. ASEX questionnaire has been validated in several studies and all of the described domains are most commonly affected adversely by age, depression [2], psychotropic drugs [3], cardiovascular diseases [4, 5], sleep apnea [6]. Finally, ASEX scale does not contain sexually explicit questions that prevent patient noncompliance or hesitation.

VQ11

Quality of life was evaluated by VQ11 which is intended to assess diverse components of quality of life (functional: 3 items, psychological: 4 items, social: 4 items), through 11 questions answered with a rating from 1 (not at all) to 5 (extremely) [7, 8]. Its validity has been compared to SGRQ and short-Form-36 [7] and VSRQ [8]. The reliability of VQ11 in COPD patients has been successfully demonstrated. Higher scores indicate less favorable quality of life. A total score greater than or equal to 22 indicates a poor quality of life, and in this case adequate to examine the 3 components of health: physical (eg, physical capacity), psychological (eg, self-esteem, and), and social (eg, social relationship, [9]).

Hospitalized Anxiety and depression (HAD)

The HAD scale determines the patient’s psychological state in terms of anxiety and depression [10]. HAD scale allows the detection of depression and/or anxiety through 14 items (each item scoring from 0 to 3), to indicate symptom frequency. Higher frequencies translating to higher scores.. Seven questions are related to anxiety and seven to depression, providing two separate scores. A score of 0-7 is considered as being in the normal range, a score of 8-10 to indicate possible diagnosis, and a score of 11 or more indicates probable presence of an altered mood.

Fatigue severity scale (FSS)

The FSS is a questionnaire with nine questions estimating the fatigue severity in different situations during the past week. Grading ranges from 1 (strong disagreement) to 7 (strong agreement) where the final score is the mean value of the nine items, and a score ≥ 4 is interpreted as fatigue [11]. FSS scale has been validated in COPD [12, 13].

STROBE Statement—Checklist of items that should be included in reports of ***cohort studies***

**COPD burden on Sexual well-being**

|  | Item No | Recommendation | COPD burden on Sexual well-being  (Page and line numbers correspond to the text with visible marks) |
| --- | --- | --- | --- |
| **Title and abstract** | 1 | (*a*) Indicate the study’s design with a commonly used term in the title or the abstract | NO |
|  |  | (*b*) Provide in the abstract an informative and balanced summary of what was done and what was found | P2 L8 -21 abstract |
| Introduction | | |  |
| Background/rationale | 2 | Explain the scientific background and rationale for the investigation being reported | p 4 L 2-17 |
| Objectives | 3 | State specific objectives, including any prespecified hypotheses | p 4 L 18-19 |
| Methods | | |  |
| Study design | 4 | Present key elements of study design early in the paper | p 4 L 23 |
| Setting | 5 | Describe the setting, locations, and relevant dates, including periods of recruitment, exposure, follow-up, and data collection | p 4 L 23 “ from march to may 2019”  p 4 L25 “data were collected at inclusion” |
| Participants | 6 | (*a*) Give the eligibility criteria, and the sources and methods of selection of participants. Describe methods of follow-up | p 4 L23-25 p5 L1-2 “On-line questionnaires were submitted on the Santé Respiratoire France website and by e-mailing list »  “Once the questionnaire was completed it was first tested then distributed to every member of association BPCO through web distribution. Then survey data was completed by volunteers, anonymously and respects the “strengthening the reporting of observational studies in epidemiology” (STROBE) statement » |
|  |  | (*b*) For matched studies, give matching criteria and number of exposed and unexposed | Not relevant |
| Variables | 7 | Clearly define all outcomes, exposures, predictors, potential confounders, and effect modifiers. Give diagnostic criteria, if applicable | All variables (questionnaires) are detailed p5-6 |
| Data sources/ measurement | 8* | For each variable of interest, give sources of data and details of methods of assessment (measurement). Describe comparability of assessment methods if there is more than one group | P6 l24-26 |
| Bias | 9 | Describe any efforts to address potential sources of bias | p12L16 – p13L10 |
| Study size | 10 | Explain how the study size was arrived at | p6 L23-25 |
| Quantitative variables | 11 | Explain how quantitative variables were handled in the analyses. If applicable, describe which groupings were chosen and why | p6 L26– p7 L1 |
| Statistical methods | 12 | (*a*) Describe all statistical methods, including those used to control for confounding | p6 L 23– p7L4 |
|  |  | (*b*) Describe any methods used to examine subgroups and interactions | Not relevant |
|  |  | (*c*) Explain how missing data were addressed | P6 L28 and Iable 1 |
|  |  | (*d*) If applicable, explain how loss to follow-up was addressed | Not relevant |
|  |  | (*e*) Describe any sensitivity analyses | No relevant |
| Results | | |  |
| Participants | 13* | (a) Report numbers of individuals at each stage of study—eg numbers potentially eligible, examined for eligibility, confirmed eligible, included in the study, completing follow-up, and analysed | “Online survey was assessed from March to May 2019 through the association Santé respiratoire France”  “Seven hundred and fifty one patients answered to the survey” |
|  |  | (b) Give reasons for non-participation at each stage | Missing data reported in Table 1 |
|  |  | (c) Consider use of a flow diagram NO | Not relevant |
| Descriptive data | 14* | (a) Give characteristics of study participants (eg demographic, clinical, social) and information on exposures and potential confounders | p 7 L8-L12  Table 1 |
|  |  | (b) Indicate number of participants with missing data for each variable of interest | Table 1 |
|  |  | (c) Summarise follow-up time (eg, average and total amount) | p 4 L23 |
| Outcome data | 15* | Report numbers of outcome events or summary measures over time | Not relevant |
| Main results | 16 | (*a*) Give unadjusted estimates and, if applicable, confounder-adjusted estimates and their precision (eg, 95% confidence interval). Make clear which confounders were adjusted for and why they were included | Tables and Figures are provided as recommended |
|  |  | (*b*) Report category boundaries when continuous variables were categorized | Not relevant |
|  |  | (*c*) If relevant, consider translating estimates of relative risk into absolute risk for a meaningful time period | Not relevant |
| Other analyses | 17 | Report other analyses done—eg analyses of subgroups and interactions, and sensitivity analyses | Not relevant |
| Discussion | | |  |
| Key results | 18 | Summarise key results with reference to study objectives | P11 L1-10 |
| Limitations | 19 | Discuss limitations of the study, taking into account sources of potential bias or imprecision. Discuss both direction and magnitude of any potential bias | p 13 L6-13 |
| Interpretation | 20 | Give a cautious overall interpretation of results considering objectives, limitations, multiplicity of analyses, results from similar studies, and other relevant evidence | p 13 L6-13 and L19-25 |
| Generalisability | 21 | Discuss the generalisability (external validity) of the study results | P13 L19-25 |
| Other information | | |  |
| Funding | 22 | Give the source of funding and the role of the funders for the present study and, if applicable, for the original study on which the present article is based | P15 L13-17 |

*Give information separately for exposed and unexposed groups.

**Note:** An Explanation and Elaboration article discusses each checklist item and gives methodological background and published examples of transparent reporting. The STROBE checklist is best used in conjunction with this article (freely available on the Web sites of PLoS Medicine at http://www.plosmedicine.org/, Annals of Internal Medicine at http://www.annals.org/, and Epidemiology at http://www.epidem.com/). Information on the STROBE Initiative is available at http://www.strobe-statement.org.

References

1. McGahuey CA, Gelenberg AJ, Laukes CA, et al. The Arizona Sexual Experience Scale (ASEX): reliability and validity. J Sex Marital Ther. 2000;26(1):25‐40.

2. Clayton AH, El Haddad S, Iluonakhamhe JP, Ponce Martinez C, Schuck AE. Sexual dysfunction associated with major depressive disorder and antidepressant treatment. Expert Opin Drug Saf. 2014;13(10):1361‐1374.

3. Lorenz T, Rullo J, Faubion S. Antidepressant-Induced Female Sexual Dysfunction. Mayo Clin Proc. 2016;91(9):1280‐1286.

4. Eyada M, Atwa M. Sexual function in female patients with unstable angina or non-ST-elevation myocardial infarction. J Sex Med. 2007;4(5):1373‐1380

5. Banerjee D, Vargas SE, Guthrie KM, et al. Sexual health and health-related quality of life among women with pulmonary arterial hypertension. Pulm Circ. 2018;8(4):2045894018788277

6. Budweiser S, Enderlein S, Jörres RA, et al. Sleep apnea is an independent correlate of erectile and sexual dysfunction. J Sex Med. 2009;6(11):3147‐3157.

7. Ninot G, Soyez F, Prefaut C. A short questionnaire for the assessment of quality of life in patients with chronic obstructive pulmonary disease: psychometric properties of VQ11. Health Qual Life Outcomes. 2013;11:179.

8. Coquart JB, Heutte N, Terce G, Grosbois JM. Convergent Validity and Minimal Clinically Important Difference of the Maugeri Foundation Respiratory Failure Questionnaire (MRF-28) and the Chronic Obstructive Pulmonary Disease-Specific Health-Related Quality of Life questionnaire (VQ11). Int J Chron Obstruct Pulmon Dis. 2019;14:2895‐2903.

9. Mahler DA. How should health-related quality of life be assessed in patients with COPD? Chest. 2000;117(2):54–57. doi:10.1378/ chest.117.2_suppl.54S).

10. Zigmond AS, Snaith RP. The hospital anxiety and depression scale. Acta Psychiatr Scand. 1983;67(6):361–370.

11. Valko PO, Bassetti CL, Bloch KE, Held U, Baumann CR. Validation of the fatigue severity scale in a Swiss cohort. Sleep. 2008;31(11):1601‐1607.

12. Economou NT, Ilias I, Velentza L, et al. Sleepiness, fatigue, anxiety and depression in Chronic Obstructive Pulmonary Disease and Obstructive Sleep Apnea - Overlap - Syndrome, before and after continuous positive airways pressure therapy. PLoS One. 2018;13(6):e0197342.

13. Kovelis D, Gomes ARS, Mazzarin C, Biazim SK, Pitta F, Valderramas S. Effectiveness and safety of supervised home-based physical training in patients with copd on long-term home oxygen therapy: a randomized trial. Chest. 2020;S0012-3692(20)30546-8.
